# Variation within the non-coding genome influences genetic and epigenetic regulation of the human leukocyte antigen genes

**DOI:** 10.3389/fimmu.2024.1422834

**Published:** 2024-09-17

**Authors:** Thilona Arumugam, Theolan Adimulam, Anmol Gokul, Veron Ramsuran

**Affiliations:** ^1^ School of Laboratory Medicine, Medical Science, University of KwaZulu-Natal, Durban, South Africa; ^2^ Centre for the AIDS Programme of Research in South Africa (CAPRISA), Durban, South Africa

**Keywords:** human leukocyte antigen system (HLA), major histocompatibility complex (MHC), single nucleotide polymorphisms (SNP), epigenetics, DNA methylation, non-coding RNA, microRNA

## Abstract

Variation within the non-coding genome may influence the regulation and expression of important genes involved in immune control such as the human leukocyte antigen (HLA) system. Class I and Class II HLA molecules are essential for peptide presentation which is required for T lymphocyte activation. Single nucleotide polymorphisms within non-coding regions of HLA Class I and Class II genes may influence the expression of these genes by affecting the binding of transcription factors and chromatin modeling molecules. Furthermore, an interplay between genetic and epigenetic factors may also influence HLA expression. Epigenetic factors such as DNA methylation and non-coding RNA, regulate gene expression without changing the DNA sequence. However, genetic variation may promote or allow genes to escape regulation by epigenetic factors, resulting in altered expression. The HLA system is central to most diseases, therefore, understanding the role of genetics and epigenetics on HLA regulation will tremendously impact healthcare. The knowledge gained from these studies may lead to novel and cost-effective diagnostic approaches and therapeutic interventions. This review discusses the role of non-coding variants on HLA regulation. Furthermore, we discuss the interplay between genetic and epigenetic factors on the regulation of HLA by evaluating literature based on polymorphisms within DNA methylation and miRNA regulatory sites within class I and Class II HLA genes. We also provide insight into the importance of the HLA non-coding genome on disease, discuss ethnic-specific differences across the HLA region and provide guidelines for future HLA studies.

## Introduction

Most diseases found in humans are associated with a 4-megabase region known as the major histocompatibility complex (MHC). This region is central to the control of most diseases as it is rich in genes involved in inflammation and immune defense (1, 2). The human MHC, also known as the human leukocyte antigen (HLA) system is sorted into three classes. Class I HLA genes consist of classical (HLA-A, HLA-B, and HLA-C) and non-classical (HLA-E, HLA-F, and HLA-G) molecules (3). Class I molecules reside on the surface of all nucleated cells and are involved in presenting endogenous self and non-self peptides to cytotoxic CD8^+^ T lymphocytes (3). On the other hand, class II molecules (HLA-DP, HLA-DQ, and HLA-DR) are generally expressed on professional antigen-presenting cells and present extracellularly derived peptides to CD4^+^ T lymphocytes (3). Lastly, class III consists of more than 60 proteins that function in inflammation, activation of the complement cascade, and cellular stress (4, 5).

As the MHC locus is the most gene-dense region, it also contains the most genetic variation. More than 16,000 HLA alleles exist which encode for approximately 13,000 protein variants when considering only classical class I and II genes (6). The thousands of alleles are named according to the Human Genome Mapping Nomenclature Committee. Each unique allele name consists of the gene name followed by an asterisk and a number of up to four digits separated by a colon. The first two digits describe the serologic assignment. The third digit describes alleles that differ in at least one synonymous substitution in an exon, while the fourth digit describes alleles that differ by a single SNP in non-coding regions. Sometimes a suffix can be added to the unique allele to indicate the state of expression: “L” (Low), “S” (secreted), “N” (null), “C” (cytoplasm), “A” (Aberrant) and “Q” (questionable) (7).

The association of HLA variants and disease risk was first established nearly 50 years ago, and since then several hundreds of ailments including cancers, infectious diseases, autoimmune disorders, and neuropathologies have been linked to the presence of certain HLA alleles (8–11). The impact of the HLA region on disease is best seen in autoimmune conditions as the HLA region, mainly class II genes, accounts for 50% of genetic predisposition to autoimmune conditions (12). For instance, the DQA1*05:01-DQB1*02:01-DRB1*03:01 (DQ2-DR3) and DQA1*03:01-DQB1*03:02-DRB1*04 (DQ3-DR4) haplotypes confer the highest risk to type 1 diabetes mellitus (13). In contrast, HLA class I associations with autoimmune conditions are less common, however, they still have a strong association with conferring disease risk such as in the case of HLA-B*27 which the strongest genetic contributors to ankylosing spondylitis in Europeans (14, 15). Furthermore, sensitivity to certain drugs has also been linked to the presence of certain HLA alleles. For example, HLA-B*57-positive individuals are hypersensitive to the anti-retroviral known as Abacavir (16, 17).

Studies have largely focused on the variation within the coding region of HLA genes as it has a direct effect on antigen presentation. The frequency of single nucleotide polymorphisms (SNPs) in non-coding regions generally exceeds those found in the coding region. Zhao et al. (2003) found that intronic and untranslated regions had a SNP density of 8.21 and 7.51 SNPs per 10 kb respectively, while exonic regions only had a density of 5 SNPs per 10 kb (18). Due to genetic variation being an underlying factor contributing to disease risk, surely non-coding HLA variants should be closely examined.

The non-coding genome is dispersed across intergenic and intronic regions. It encompasses the UTRs, promoters, and distal regulatory elements. The 5’ and 3’ UTR flank the start and end of a gene and regulate protein levels by influencing translation, mRNA stability, and secondary structure (19, 20). The promoter regions were found ~0.5kb from the transcriptional start site. Theyact as a scaffold for transcriptional machinery such as transcription factors and RNA polymerases. Using enhancers and silencers found up to 1 megabase away from promoters, transcription factors can either initiate or suppress transcription. Non-coding SNPs typically regulate expression by influencing transcription factor binding, long-range chromosomal interactions, and mRNA stability (21). The induced changes in HLA expression can significantly impact antigen processing and presentation, thereby affecting the peptide repertoire presented to T cells and consequently altering T cell priming capacity. For instance, reduced HLA expression could result in decreased presentation of antigenic peptides to T cells and inadequate T cell activation. This diminishes the recognition and elimination of infected or cancerous cells by the immune system. Alternatively, higher HLA expression could enhance antigen presentation, broaden the peptide repertoire, and improve T cell priming capacity, leading to more effective immune responses (22). Differential expression of HLA genes and proteins has been associated with various diseases (23–26). Through its influence on gene expression, non-coding genetic variants may be a contributing factor to these diseases.

Genetic variation in non-coding regions may also influence gene expression through epigenetic mechanisms. Epigenetic mechanisms generally target non-coding regions and can influence gene expression without modifying DNA sequences. These factors include DNA methylation, histone modifications, and non-coding RNAs such as microRNAs and long non-coding RNAs (27). A few studies have identified epigenetic factors responsible for controlling HLA expression. For instance, Ramsuran et al. (2015) found that DNA methylation of *HLA-A* promoters is a major factor driving allele-specific expression of HLA-A (28). Kulkarni et al. (2013) also found that the 3’UTR of *HLA-C* contains binding sites for miR-148, resulting in lower HLA-C expression in individuals with Crohn’s disease (23). Polymorphisms found within these DNA methylation and non-coding RNA binding sites may alter the epigenetic regulation of these genes. It is therefore crucial to explore the interaction between genetic and epigenetic regulatory mechanisms.

In this review, we discuss variants found within the class I and class II HLA non-coding genome and the association it may have with disease outcomes. We further explore the link between genetic variation and its influence on epigenetic factors controlling HLA expression, the importance of the non-coding genome on disease, discuss ethnic specific differences across the HLA region, and provide guidelines for future HLA studies.

## Genetic non-coding variants affecting *HLA* expression

Most studies have evaluated coding variations of *HLA* as they could lead to amino acid substitutions in the protein sequence, affecting protein structure, stability, antigen binding specificity, or interactions with other molecules involved in antigen presentation. The magnitude of change in HLA expression resulting from coding SNPs can also vary, depending on factors such as the location and nature of the amino acid substitution, and its impact on HLA expression and function (29). Non-coding variants have been shown to play a role in the regulation of both class I and II genes. Mutations in the non-coding genome can influence gene expression by affecting transcription factor binding, influencing long-range chromatin interactions, and modulating mRNA stability (21). It may also influence protein expression by altering post-transcriptional processes such as splicing, polyadenylation, cleavage, ribosome binding, and assembly. They also have greatly effect gene expression. for instance, rs2395471 and HLA-C 263 insertion/deletion polymorphisms account for 40% variation in HLA-C expression (23, 30).

A genome-wide association study (GWAS) conducted on Malaysian Chinese patients with nasopharyngeal carcinomas detected a significant association between certain SNPs found in the *HLA-A* gene and either susceptibility or resistance to nasopharyngeal carcinomas (31). Rs41545520 (G>T), which is found in the promoter region of *HLA-A* may affect *HLA-A* transcription as the G allele creates a binding site for activating transcription factor 3 (ATF3). Through its interaction with the cyclic adenosine monophosphate response element (CRE) at gene promoters with TGACATCA motifs, ATF3 represses transcription (32, 33). Using the Genevar Database, the authors found that rs41545520 (G>T) is in strong linkage disequilibrium with rs2860580 (A>G), an HLA-A intronic variant (31), which was previously associated with nasopharyngeal carcinomas (34, 35). Expression quantitative trait locus (eQTL) analysis using Genevar Database showed that the wild type G allele is associated with stronger binding with ATF3 and thus lower *HLA-A* expression. However, the mutant T allele is associated with higher *HLA-A* expression, due to its weaker ATF3 binding affinity (31). Furthermore, the T allele (rs41545520) marks a higher expression of the protective HLA-A*11:01 allele in nasopharyngeal carcinoma. The higher HLA-A expression allows for increased presentation of tumor-specific antigens to cytotoxic T cells and improved clearance of the tumor cells (31). Allelic polymorphisms have also been associated with higher expression of HLA-A*31 and HLA-A*33 alleles. Two polymorphisms, rs41272547 (C>A) found in the 5’UTR and rs1061235 (C>T) in the 3’UTR were only found in HLA-A*31 and HLA-A*33 alleles. The mutant alleles were associated with significantly higher *HLA-A* expression in heterozygous HLA-A*31 and HLA-A*33 individuals (36). Prediction analysis demonstrated that rs41272547 may disrupt a nuclear factor kappa B (NF-kB) and activating protein 2 (AP2) binding motifs and may be responsible for creating a site for glucocorticoid receptor binding. Transcriptional activity was assessed using luciferase reporter assay which found that the mutant A allele was associated with higher transcriptional activity compared to the reference allele (C) (36). While rs41272547 may affect transcriptional regulation, rs1061235 may affect post-transcriptional regulation of HLA-A. The consensus sequence (which contains a C allele for rs1061235) contains binding sites for miRNA binding (discussed later on) (36). Rs9260118 (T>C) and rs9260119 (T>A) were also found to reside in the 5’UTR. Wild type alleles were associated with significantly higher expression of HLA-A*01/A*11/A*03/A*30 alleles compared to the mutant alleles; however, these SNPs were not associated with altered transcription factor binding (36). These SNPs may be in linkage disequilibrium with additional functional SNPs. Although HLA-B is regarded as the most polymorphic gene region, consisting of approximately 3,000 SNPs, the non-coding region of HLA-B remains understudied as no polymorphisms within the non-coding region were shown to associate with HLA-B expression or disease. Furthermore, minor differences in the mRNA expression of *HLA-B* was reported across different *HLA-B* alleles suggesting that *HLA-B* non-coding SNPs may not play a significant role in HLA-B regulation (37, 38).

HLA-C has the lowest genetic diversity amongst the major class I genes. Like HLA-A, HLA-C is expressed in an allele-specific manner. HLA-C surface protein expression was imputed for 228 individuals from the 1000 Genomes study who were previously HLA-typed (30). Out of the 68,726 SNPs across the MHC region, rs2395471 (A>G) and, rs2249741 (A>G), were strongly associated with HLA-C imputed eQTL and surface expression. Rs2395471 is found 800 base pairs upstream of the core promoter. The association of rs2395471 and HLA-C surface expression was validated in two independent cohorts. Rs2395471 accounted for 36% variation in surface HLA-C expression in European Americans and a 28% variation in African Americans (30). A separate study supported the association of rs2395471 with HLA-C levels in peripheral blood mononuclear cells (PBMCs) from 273 European participants. Using the Alibaba prediction tool, Vince et al. (2016) found that rs2395471 may be located within a binding site for the Pit-Oct-Unc (POU) transcription factor family; however, rs2249741 did not seem to overlap with any transcription factor binding motifs (30). Electrophoretic mobility shift assays on the nuclear extracts obtained from HeLa and Jurkat cell lines as well PBMCs from healthy individuals found that rs2395471 only affects the binding of Oct1 and no other POU family members. This suggests that the G allele may account for lower *HLA-C* expression. The results were further validated by the use of luciferase reporter assays. High expressing HLA-C*01:02 and HLA-C*04:01 carry the A allele for rs2395471, while the G allele is present in low expressing HLA-C*03:04 and HLA-C*08:02. Substitution of A with G in high expressing alleles resulted in similar promoter activity of low expressing HLA-C alleles. However, the converse did not occur when the G was substituted for an A in low expressing HLA-C*03:04 and HLA-C*08:02; suggesting an additional regulatory factor may dominate over Oct1 transcriptional regulation in HLA-C*03:04 and HLA-C*08:02 (30). While the promoter activity of HLA-C*04:01 and HLA-C*08:02 differ drastically in HeLa and Jurkat cells; they are expressed at similar levels in CD3^+^ cells (39). Post-transcriptional regulatory mechanism may account for this discrepancy as HLA-C*04:01 contains a binding motif for miR-148a resulting in the degradation of HLA-C*04:01; however, HLA-C*08:02 contains a SNP which allows it to escape miR-148a binding (24). Not only does Oct1 stimulate gene transcription, it also interacts with other transcription factors such as NF-κB. Rs2395471 is located approximately 651 bp downstream of enhancer κB element in the *HLA-C* promoter. Rs2524094 (G>A) was found to disrupt enhancer sequence and led to cells being unresponsive to NF-κB stimulatory cytokines (TNF-α, IL-17A and IL-22). It would be interesting to note whether disruption of the Oct1 binding site may influence NF-κB binding at the enhancer κB element (40).

Unlike classical HLA genes, HLA-G functions through mediating immune tolerance rather than stimulation via antigen presentation. High levels of HLA-G can exert inhibitory functions against immune cells such as natural killer (NK) cells, T lymphocytes, and antigen-presenting cells allowing for successful maternal-fetal interactions and organ transplantations. Thus, polymorphisms causing low levels of HLA-G expression are more likely to result in spontaneous abortions, preeclampsia, and transplant rejection (41, 42). A 14bp insertion/deletion polymorphism found within the 3’UTR is the most well-studied HLA-G non-coding variant (42). The presence of the 14 bp insertion usually results in lower expression of circulating and membrane-bound HLA-G; while deletion of the sequence results in elevated HLA-G levels (43–46). The 14-bp insertion variant is related to the alternative splicing of the *HLA-G* primary transcript, which results in a more stable mRNA, but this higher stability does not compensate for the lower HLA-G expression. Although the presence of the 14bp sequence causes lower *HLA-G* expression, the spliced isoform that is formed is more stable than the isoforms where the 14bp sequence is removed (47). The presence of the insertion alleles has been associated with a higher risk of spontaneous abortions, preeclampsia, transplant rejections, and auto-immune conditions such as multiple sclerosis, and Crohn’s disease (43–46).

Non-coding variants also affect the expression of class II genes: HLA-DRB1, HLA-DQA1, HLA-DPA1, and HLA-DPB1 (48–51). SNPs within class II HLA genes strongly influence an individuals to risk of Hepatitis B virus (HBV) infection and viral clearance in Sichuan Han, non-Hispanic European, and Indonesian populations (49–55). Rs3077A (HLA-DPA1), rs9277535A (HLA-DPA1), and rs3135021A (HLA-DPB1) were observed at a higher frequency in healthy control than HBV carriers. The GA and AA genotypes of rs3077 were also associated with spontaneous clearance of HBV. The protective nature of rs3077A, rs9277535A, and rs3135021A could be due to changes in HLA expression. rs3077A and rs9277535A carriers have higher *HLA-DPA1* expression. The increased expression of *HLA-DPA1* may increase antigen presentation and T-cell priming allowing for protection against HBV. The mechanism behind the altered expression is unknown, however, rs3077A (HLA-DPA1), and rs9277535A are found within the 3′ UTR and may be regulated by miRNA (49–51).

**Table 1** provides a list of non-coding variants affecting the expression of class I and class II genes and how they may be associated with certain disease outcomes. These studies demonstrate that disease phenotypes are not only a product of variation in the coding region but also changes in expression through non-coding genetic variants. While hundreds of non-coding variants exist, only a handful of studies have found a direct link between these variants and expression, Furthermore, even fewer studies have investigated the functional relevance of these polymorphisms.

**Table 1:** Genetic non-coding variants which may associate with *HLA class I* and *II* expression.

Gene	SNP	Mechanism	Disease	Ref
*HLA-A*	rs41545520 (G>T, 5’UTR)	G allele forms a predictive binding site for the transcriptional repressor, ATF3 which leads to reduced *HLA-A* expression. ATF3 binding site is lost in the presence of the T allele resulting in higher *HLA-A* expression. T allele is protective against nasopharyngeal carcinoma	Nasopharyngeal carcinoma	(31)
*HLA-A*	rs9260102 (G>T, 2KB, Upstream)	Functional analysis showed T allele creates an IRF1 binding site. Unknown whether HLA-A expression is affected.	N/A	(56)
*HLA-A*	rs12202296, (T>C, 2KB upstream)	T allele is associated with lower *HLA-A* expression than the C allele. C allele denoted significant susceptibility towards cold medicine-related Stevens–Johnson syndrome and toxic epidermal necrolysis due to the high expression of *HLA-A*.	Stevens–Johnson syndrome/toxic epidermal necrolysis	(57)
*HLA-A*	rs41272547 (C>A, 5’UTR) and rs1061235, (C>T, 3’UTR) rs9260118 (T>C, 5’UTR) and rs9260119 (T>A, 5’UTR)	Mutated A allele (rs41272547) and T allele (rs1061235) is strongly associated with higher expression of HLA-A*31 and A*33 alleles, in comparison to the consensus sequence. The presence of the A allele (rs41272547) disrupts NF_-K_B and AP-2 binding sites and creates a glucocorticoid receptor binding site. The presence of wild-type T alleles for rs9260118 and rs9260119 are associated with significantly higher expression of HLA-A*01/A*11/A*03/A*30 alleles compared to the presence of alternative C and A alleles respectively.	N/A	(36)
*HLA-C*	rs2395471, (G>A, 2KB upstream variant)	The A alleles have a high affinity for Oct1 binding resulting in higher HLA-C expression whereas the G allele has a low affinity for OCT1 binding resulting in low *HLA-C* expression	N/A	(30)
*HLA-C*	rs2524094 (G>A, 2KB upstream variant) rs10657191 (AGAAG> AGAAGAAG, 2KB upstream variant)	HLA-Cw*0602 is a psoriasis risk allele. rs2524094 associated with allele-specific expression of *HLA-C*. A allele is displayed in HLA-C*0602 which results in lower promoter activity at enhancer κB element, and thus, responds weakly to TNF-a, IL-17 and IL-22 stimulation HLA-Cw*0602 also contains a 3bp deletion (rs10657191) in the interferon response stimulated element (ISRE) resulting in the non-responsiveness to IFNs.	Psoriasis	(40)
	rs9264942 (T>C, 5’UTR)	C allele is associated with higher expression and associated with lower mean viral loads and progressed slower to AIDS compared to individuals with and T allele	HIV	(25)
*HLA-C*	rs9264942 (T>C, 5’UTR) rs2395471 (G>A, 2KB Upstream Variant)	Higher frequency of CC and CT genotypes in individuals with ulcerative colitis but it did not affect the development of Crohn’s disease. *HLA-C* expression on the surface of CD3e^+^CD8a^+^ T lymphocyte, neutrophils, and macrophages were higher than TT Individuals with AA or AG genotypes had higher expression than those with GG genotypes in CD3e^+^CD8a^+^ T lymphocyte; however expression was not affected in macrophages and neutrophils	Inflammatory bowel disease	(58)
*HLA-G*	rs1233335 (G>A, 2KB Upstream Variant), and rs915670 (C>G, 2KB Upstream Variant)	Presence of mutant A (rs1233335) and G/T (rs915670) alleles resulted in lower HLA-G expression in individuals with idiopathic recurrent spontaneous abortions compared to controls.	Idiopathic recurrent spontaneous abortions	(59)
*HLA-G*	rs9380142 (A>G, 3’UTR)	G allele associated with reduced HLA-G expression. rs9380142 is located near an AUUUA motif and increases the rate of *HLA-G* mRNA degradation. AA and AG genotypes are associated with preeclampsia.	Pre-eclampsia	(43)
*HLA-G*	rs371194629 (ATTTGT>ATTTGTTCATGCCT, 3’UTR)	Individuals with insertion polymorphism had significantly higher mean serum HLA-G levels and were significantly associated with fewer episodes of cellular rejection.	Transplant rejection	(44)
		14bp insertion polymorphism results in higher expression of membrane-bound HLA-G expression due to the higher HLA-G mRNA stability compared to the 14pb deletion allele.		(45)
		High serum and CSF HLA-G levels in individuals with deletion polymorphism	Multiple sclerosis	(60)
		The 14-bp Del/Del and 14-bp Del/Ins genotypes are associated with high *HLA-G* expression. The 14-bp Del/Ins polymorphism is associated with an increased risk of Crohn’s disease	Crohn’s disease	(46)
*HLA-DRB1/* *HLA-DQA1*	rs9271597 (T>A), rs9271600 (T>G), and rs9271601 (A>T)	The mutant alleles in the haplotype are associated with elevated eQTL for *HLA-DRB1* and *HLA-DQA1* Predictive analysis shows that rs9271597 possibly alters E2F and Sox3 binding; rs9271600 may affect binding of Cdx2, Dbx1, Foxa, HDAC2, Hoxa5, Lhx3, Mef2, Ncx, Pou1f1, Pou2f2, Pou5f1, Sox19, Sox2, Sox5, and Zfp105; and rs9271601 could possibly affect the bind motifs for Cdx2, Dbx1, Foxa, GR, Hoxa5, Lhx3, Ncx, Sox19, Sox2, Sox5, and Zfp105.	Vitiligo	(48)
*HLA-DPA1*	rs3077 (A>G, 3’UTR)	The presence ofGA and AA alleles corresponded with reduced *HLA-DPA1* expression which in turn is associated with greater risk susceptibility to HBV infection.	HBV	(49)
*HLA-DPA1*	rs2395309 (A>G), rs3077 (A>G, 3’UTR) and rs2301220 (C>T, Intron)	rs2395309, rs3077 and rs2301220 are in strong LD in Europeans and strongly associated with *HLA-DPA1* expression. Further analysis of rs3077 showed that GG genotypes were associated with lower expression for *HLA-DPA1* and a greater risk of chronic HBV.	HBV	(50, 51)
*HLA-DPA1*	rs1431403 (T>C, Intron variant)	Individuals with CC genotype had lower *HLA-DPA1* expression compared to CT and TT genotypes. However, both CC and TT were associated with a higher risk of anembryonic pregnancy than CT genotype. Prediction analysis found that the T allele formed an interferon consensus sequence binding site.	Anembryonic pregnancy	(52)
*HLA-DPA1*	rs9277341 (T>C, Intron)	The T allele is associated with higher *HLA-DPA1* expression in contrast to the C allele which is associated with lower *HLA-DPA1* expression across multiple brain regions.	Psychiatric Disorders	(53)
*HLA-DPB1*	rs9277535 (A>G, 3’UTR)	GG genotype was strongly associated with both increased risk of chronic hepatitis B and decreased expression of *HLA-DPB1*	HBV	(50)
*HLA-DPB1*	rs9277535 (A>G, 3’UTR)	*HLA-DPB1* expression was significantly reduced in the presence of GG genotype compared to AA genotype.	Rheumatoid arthritis	(54)
*HLA-DQA1*	rs2187668 (C>T, Intron), rs28383345 (G>A, 5’UTR), rs36173887 (A>G, upstream variant), and rs114929610 (C>A, upstream variant)	Variants in the promoter region of *HLA-DQA1* were strongly associated with idiopathic membranous nephropathy in the Chinese Han population. Using RegulomeDB rs72848263, rs114929610, rs36173887, rs115222936, and rs28383345 could affect the binding motifs of transcription factors. Rs28383345 is found in the open reading frame of *HLA-DQA1* and may affect translation.	Idiopathic membranous nephropathy	(55)

## Linking genetic variation with epigenetic regulation of *HLA* genes

Genetic variation has long been regarded as a major driver of disease-associated phenotypic variation. The contribution of epigenetic modifications to disease is becoming more recognized. Genetic and epigenetic changes are often studied independently, however, sometimes they may interact. SNPs can influence epigenetic mechanisms by affecting DNA methylation sites and altering non-coding RNA-target binding affinities. This in turn affects gene expression or results in the preferential expression of a specific allele (61). In the subsequent sections, we discuss the implications of genetic variation on DNA methylation and miRNA-mediated regulation of HLA expression.

## Polymorphic methylation sites affecting HLA regulation

DNA methylation is the best characterized epigenetic modification. In mammals, methylation usually occurs on a cytosine base that is 5’ to a guanine base (CpG). This generally inhibits gene transcription by promoting a heterochromatin state and preventing transcription factor binding in the promoter regions of genes (62). Regulation of *HLA* genes is no exception to this phenomenon. Thus, methylation of HLA class I and II promoter regions are usually associated with reduced *HLA* mRNA expression (62–64). Promoter methylation of essential transcriptional HLA-regulating components such as the class II transactivator (CIITA) may also influence HLA expression (65, 66).

**Figure 1:**
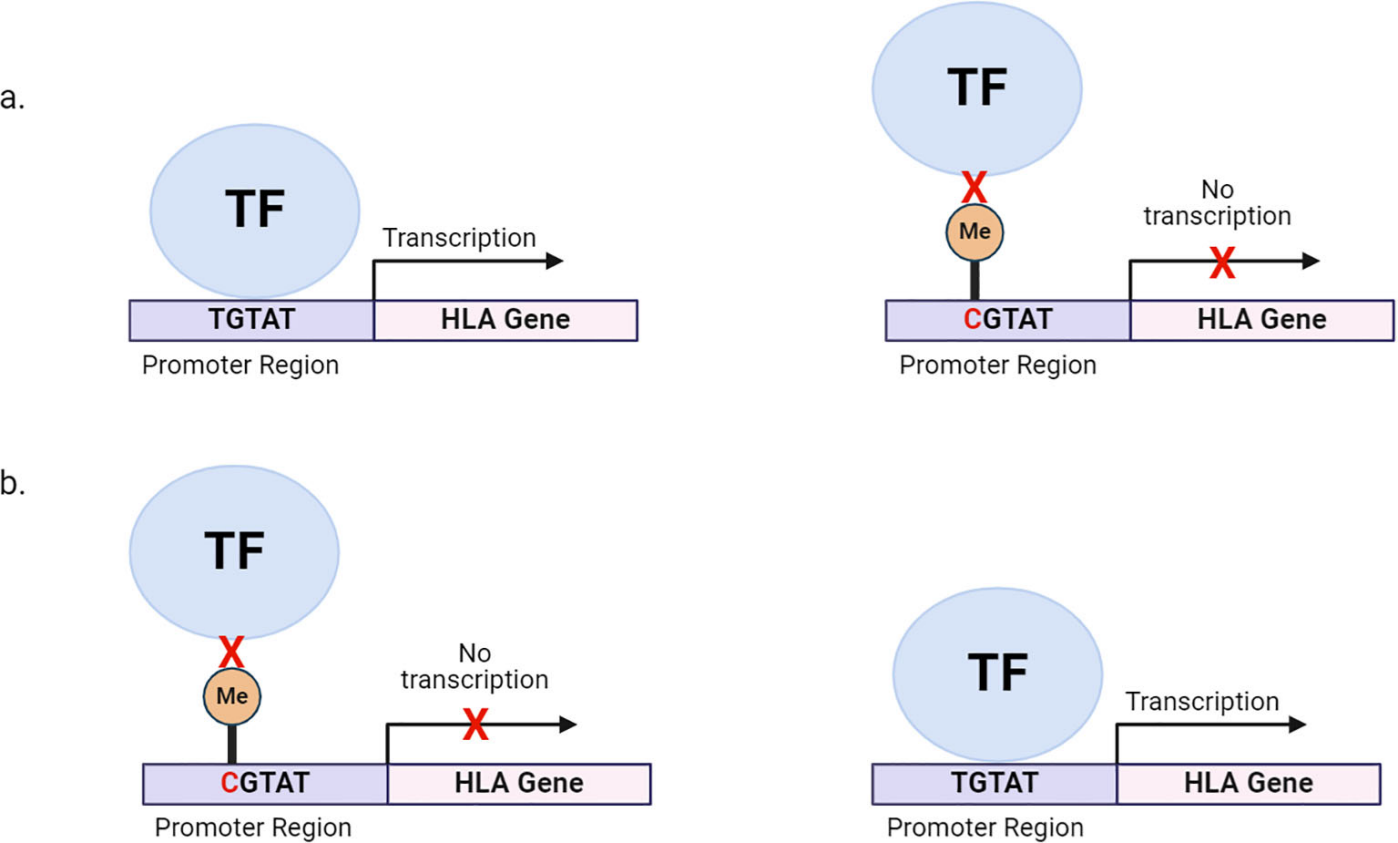
Genetic variation within HLA promoter regions can alter DNA methylation states, affecting HLA expression. **(A)** SNPs within the promoter can also create a CpG site. Methylation of CpG sites, preventing the binding of transcription factors and other chromatin factors and leading to reduced HLA transcription. **(B)** SNPs within the promoter can destroy CpG site, allowing for the binding of transcription factors and other chromatin factors and leading to HLA transcription. TF, transcription factor; Me, methyl group.

Genetic variants modulate quantitative changes in methylation levels at specific loci. This is known as methylation quantitative trait loci (meQTL) (67). meQTLs can affect a few CpG sites or influence the methylation of multiple CpG sites distributed across the extended genome and are often associated with changes to gene expression levels. SNPs usually underlie meQTLs (67, 68). SNPs can either introduce or abolish CpG sites leading to drastic changes in methylation at single CpG sites. For instance, a C-to-T transition on ‘C’ of CpG dinucleotides leads to a loss of a CpG site, resulting in a loss of methylation and an increase in gene expression (**Figure 1**) (69). Furthermore, these methylation-associated SNPs (meSNPs) may affect the methylation status of neighbouring CpG sites or influence expression through high linkage disequilibrium (69) (**Table 2**).

**Table 2:** Polymorphic methylation sites affecting HLA regulation.

Gene	SNP	Mechanism	Disease	Ref
*HLA-A*	Allele-specific methylation	*HLA-A* expression differs in an allele-specific manner in accordance with DNA methylation levels in the European population. SNPs within high expressing HLA-A*24 disrupts a CpG site found in the promoter region. CpG site is retained in low expressing HLA-A*03 allele.	N/A	(28)
*HLA-C*	Allele-specific methylation	Cg03216697, cg01521131, cg09382842, and cg09556042 are only observed in the HLA-C*07 allele and are associated with higher levels of DNA methylation and gene expression of the *HLA-C* gene in patients with endometriosis compared to healthy controls.	Endometriosis	(70)
*HLA-G*	−725(C>G, Promoter)	The mutant G allele creates a CpG site. This disrupts an IRF1 binding site and induces promoter methylation. However, there was no association in individuals with recurrent spontaneous abortions.	Miscarriage	(71, 72)
*Class II risk haplotypes*	Allele-specific methylation	metQTLs for HLA class II and genes associated with type 1 diabetes.	Diabetes	(73)
*HLA-DQA2*	rs6906021 (T>C, 2KB Upstream Variant)	The introduction of the C allele creates a CpG site. Methylation of the CpG site may result in lower *HLA-DQA2* expression.	Asthma	(74)
*HLA-DQB1*	rs1063355 (C>A, 2KB Upstream Variant)	CC is associated with higher methylation of cg22984282, while CA and AA are associated with lower expression and are shown to influence *HLA-DQB1* expression in pancreatic islets.	Type 2 diabetes	(75)
*HLA-DRB1 and HLA-DQB1*	rs9275596 (C>T, Intergenic variant)	meQTL SNP, rs9275586 significantly alters the methylation of cg15982117 and cg18024368 in *HLA-DRB1* and *HLA-DQB1* genes and is strongly associated with the risk of peanut allergies	Peanut allergy	(76)
*HLA-DRB1/HLA-DRB6*	rs9271573 (A>C, Intergenic variant)	rs9271573 (*HLA-DRB6*) is a meQTL SNP that affects methylation of cg08845336 which regulates *HLA-DRB1* expression.	Congenital heart disease	(77)
*HLA-B27*	Allele-specific methylation	Hypomethylation within HCP5 involves a CpG site that contains SNP in linkage disequilibrium with HLA-B*27 and that controls DNA methylation at this locus in an allele-specific manner.	Ankylosing spondylitis	(78)

Individual genotypes at a given locus may result in different DNA methylation patterns and may influence the levels of expressed alleles. This is known as allele-specific methylation. Allele-specific methylation is common in genetic imprinting; where the inactive gene is significantly more methylated than the actively expressed gene (79). Ramsuran et al. (2015) observed allele-specific methylation in HLA-A (28). The group found several CpG sites including a CpG island within the promoter, and another spanning intron 1 to exon 3 of *HLA-A*. Deep-sequencing of a ∼300 bp DNA fragment upstream of the *HLA-A* transcription start site found that individuals homozygous for HLA-A*03 (a low expressing linage) had one or more individual CpG sites that were methylated within the promoter region. In contrast, individuals homozygous for HLA-A*24 (a high expressing lineage) had only one methylated CpG in the promoter region (28). Interestingly, a SNP within the HLA-A*24 lineage resulted in the loss of one CpG site reducing the number of potential methylation sites and potentially affecting HLA-A*24 expression. HLA-A allelic expression may also differ due to the methylation patterns within transcription factor binding sites. Ramsuran et al. (2015) observed two methylated CpG sites within the HLA class I regulatory complex (CRC) transcription factor binding site in the HLA-A*03 lineage; however, no methylation was observed in the CRC binding motif for HLA-A*24 (28). Methylation of the CRC motif probably prevented CRC from binding to the *HLA-A* promoter, hindering HLA-A*03 transcription while transcription of HLA-A*24 continued uninterrupted as methylation did not affect CRC binding (28). The HLA-A*24 promoter contains a polymorphism that disrupts a methylation site (CpG → TpG), while the CpG site remains in lower expressed HLA-A*03 lineage, arguing for methylation-mediated suppression of expression of the HLA-A locus (Ramsuran et al., 2015).

Zhao et al. (2023) evaluated the influence of DNA methylation patterns on women with endometriosis. Fifteen CpG sites were found to be differentially methylated between women with endometriosis and healthy controls. Five of these CpG sites were found in intron seven on HLA-C and four (cg03216697, cg01521131, cg09382842, and cg09556042) were found exclusively in HLA-C*07 allele (70). Higher methylation of intron 7 also led to higher expression of HLA-C*07 in women with endometriosis. It is possible that a silencer may be present on intron 7, and that the hypermethylation may inhibit the silencer resulting in increased expression of HLA-C*07.Furthermore, HLA-C*07 is a ligand for inhibitory KIR signaling of natural killer cells, which normally clear the endometrial tissue. It is possible that the higher expression of HLA-C*07 due to hypermethylation of intron seven, resulted in the silencing of natural killer cells, thus promoting the occurrence of endometriosis (70).

Allele-specific methylation has also been noted for HLA-G; however, the role non-coding variants may play has yet to be uncovered (80). Nevertheless, variation within the *HLA-G* promoter region has been shown to influence fetal loss (71). HLA-G plays an important role in embryo implantation, and fetal development as well as in protecting the fetus from the maternal immune system (81). Thus, it is not surprising that loss of HLA-G may result in adverse pregnancy outcomes such as preeclampsia, unsuccessful embryo implantation, and fetal loss (81, 82). Variation of the *HLA-G* promoter region was investigated within 42 Hutterites from South Dakota (71). Hutterites have a naturally high fertility rate despite the high level of consanguineous relationships within the population (83). Eighteen SNPs were identified in a 1,300bp region upstream of the *HLA-G* transcriptional start site. Only one (-725C>G) of these eighteen polymorphisms was associated with significant fetal loss. The presence of the -725G allele in both parents was associated with a significant risk of miscarriage (71). The G allele at -725 creates a CpG site at -726 and -275 which is located approximately 10bp from an IRF1 binding site. The presence of the G allele at -275 promoted the methylation of the C nucleotide found at -726. This may have disrupted IRF1 binding resulting in lower *HLA-G* expression. Low HLA-G expression leads to increased maternal immune responses against the fetus and impaired vascular remodeling of the placenta contributing to miscarriageand thus promoting fetal loss (71). However, in a study conducted on couples who had recurrent spontaneous abortions, no methylation was observed at -276C in the presence of the G allele at -275 and there was no association between the -275 SNP and recurrent spontaneous abortions (72). This difference in results may be attributed to the different study groups used. The first study used couples who suffered from miscarriages but also had pregnancies that were carried to full term, while the latter study focused on couples who had recurrent miscarriages.

Polymorphic methylation sites have also been noted in class II HLA genes. Several GWAS studies have identified SNP-CpG sites located in *cis*-regions of *HLA-DQ* and *HLA-DR*. These SNP-CpG sites were found to be associated with asthma, type 2 diabetes, food allergies, and congenital heart disease (74, 76, 77). A GWAS conducted on the airway epithelium of asthmatic individuals found that 59% of SNPs regulated *cis*-gene expression. Of that 59%, 89.9% of those SNPs mediated *cis*-gene expression via *cis*-methylation. rs6906021 (T>C), was one of the SNPs found to regulate *HLA-DQA2* expression through methylation (74). Individuals homozygous for TT showed higher methylation for cg22933800 compared to individuals heterozygous for TC and homozygous for CC. The level of cg22933800 methylation directly correlated with *HLA-DQA2* expression. Higher expression of HLA-DQA2 can increase asthma susceptibility by enhancing antigen presentation and promoting immune responses that drive allergic inflammation and hyperactivity of the immune cells within the airways (74).

The first GWAS of well-defined food allergies was conducted on 2,759 US participants and replicated in 2,197 participants of European ancestry (76). The study found that rs9275596 (C>T) an intergenic SNP found between (*HLA-DQB1/HLA-DQA2*) showed the most significant association with peanut allergies. The study showed that rs9275596 was a meQTL for the *HLA-DRB1* and *HLA-DQB1* genes, and that methylation was associated with risk of peanut allergies (76).

In addition to peanut allergies, variation in *HLA-DR* may also harbor a risk of congenital heart disease. rs9271573 (A>C), located near *HLA-DRB6*, was found to affect the methylation of cg08845336 located on *HLA-DRB1* (77). Individuals with CC genotypes showed elevated methylation levels compared to individuals heterozygous for CA and homozygous for AA. Further, the SNP was found to colocalize with *HLA-DRB1* expression (77).

Occasionally, CpG-SNPs can be in high linkage disequilibrium with other polymorphisms, making the altered methylation state a tag for other genetic polymorphisms (9). In human pancreatic islets, rs1063355 (C>A) was found to associate with HLA-DQB1 expression (75). Individuals who presented with CC genotypes had significantly higher levels of methylation at cg22984282 than individuals with CA or AA genotypes The study found that rs1063355 was found to be in linkage with rs9272346, (2KB upstream of HLA-DQA1) a variant which is associated with increased risk of type 1 diabetes (75). Similarly, Coit et al. (2019) set out to identify differentially methylated CpG sites in HLA-B*27-positive individuals with ankylosing spondylitis (78). The CpG site cg17616250 was the most hypomethylated in ankylosing spondylitis patients relative to the osteoarthritis control group (78). Furthermore, HLA-B*27 positive patients with ankylosing spondylitis had lower methylation at this CpG site compared to HLA-B*27 negative patients (78). However, cg17616250 is located in *HLA Complex P5 RNA* (*HCP5*). The methylation status of cg17616250 is determined by the SNP rs114212906 (C>T). Carriers of the CC genotype had higher methylation levels than CT and TT carriers as the minor T allele tends to disrupt the CpG site. Rs114212906 is in strong linage disequilibrium with rs4349859, a SNP often associated with HLA-B*27 positive ankylosing spondylitis patients (78). **Table 2** summarizes studies investigating polymorphic methylation sites affecting HLA regulation.

## Non-coding variants affecting miRNA regulation of class I and II *HLA* expression

**Figure 2:**
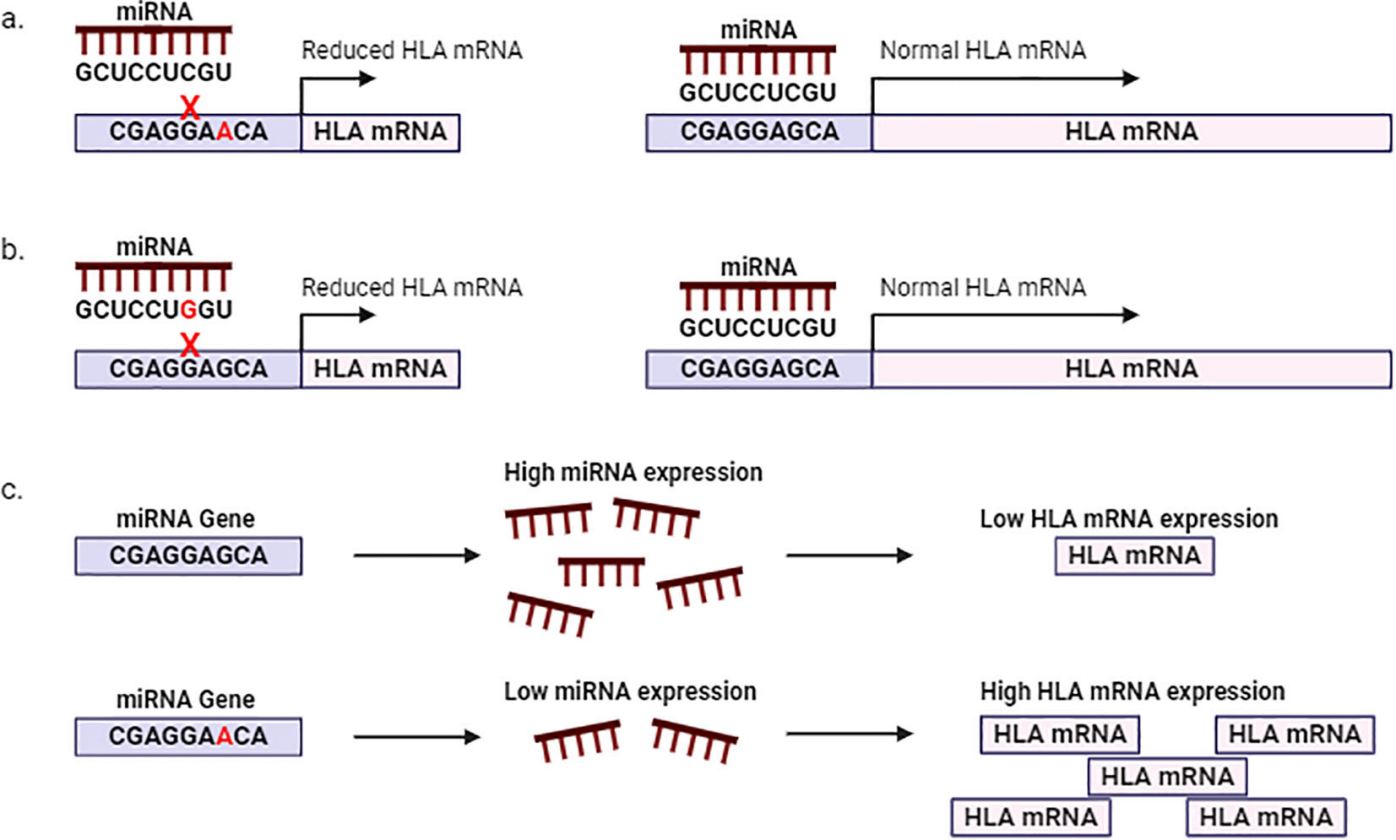
Polymorphisms affecting miRNA regulation of HLA mRNA. SNPs within the 3’UTR of HLA **(A)** or within the seed region of the miRNA **(B)** affect miRNA-HLA mRNA interactions. **(C)** SNPs within regulatory regions of the miRNA may affect miRNA expression and subsequently HLA expression.

Non-coding transcripts such as miRNAs play an essential role in regulating normal physiological processes and consequently human health and disease (84–86). MiRNAs belong to a class of small endogenous non-coding RNA molecules of approximately 20-24 nucleotides, with regulatory effects on cellular processes. Generally, a specific sequence within the 5’UTR of the miRNA, known as the “seed region” complementary base pairs with the 3’UTR of the miRNA-target transcript or mRNA. This interaction results in translational silencing and decay of the target transcript (87). The complementary relationship between miRNA-mRNA is evolutionarily conserved; however certain factors can disrupt miRNA-mRNA interactions. Genetic variation within the “seed region” of the miRNA or the 3’UTR of the targets may create, disrupt, or alter the affinity of miRNA-mRNA interactions, affecting the expression of the mRNA target and subsequently resulting in altered physiological processes or disease onset (87). Apart from affecting miRNA-mRNA interactions, SNPs within the miRNA gene may affect miRNA biogenesis or maturation, altering miRNA expression which may also affect the regulation of the target mRNA (**Figure 2**) (87, 88).

A few studies have noted genetic variation affecting miRNA-*HLA* interactions (**Table 3**). We previously discussed a study by Rene et al. (2015) which found 5’UTR and 3’UTR SNPs involved in the allele-specific regulation of *HLA-A* (36). The presence of specific polymorphisms within the 3’UTR of certain *HLA-A* alleles may contribute to allele-specific regulation of *HLA-A* expression (36). Individuals with a single HLA-A*31 or HLA-A*33 allele tend to have high HLA-A levels. The polymorphism rs1061235 (A>T) is found in the 3’UTR of *HLA-A* (36). The A allele is the predicted target for the binding of miR-526b, -609, -1290, -342-5p, and -542-5p. While the T allele which is found in HLA-A*31 and HLA-A*33 disrupts the binding of miR-526b, -609, -1290, -342-5p, and -542-5p and instead creates predicted binding sites for miR-520f and miR-651. However, the miR-520f and miR-651 may not experimentally target HLA-A*31 and HLA-A*33 as the T allele is associated with higher *HLA-A* levels (36).

**Table 3:** Genetic variants associated with miRNA-mediated regulation of HLA expression.

HLA	SNP	Mechanism	Disease association	Ref
*HLA-A*	rs1061235 (A>T, 3’UTR)	T allele disrupts binding of miR-526b, -609, -1290, -342-5P and -542-5p in HLA-A*31 and HLA-A*33, resulting in higher *HLA-A* levels.		(36)
*HLA-C*	rs67384697 (G>T; 3’UTR of HLA-C)	T allele is found in high expression *HLA-C* alleles as it disrupts miR-148a regulation; while the G allele is present in low expressing *HLA-C* alleles as it allows for miR-148aregulation.	HIV	(24)
*HLA-C*	rs735316(G>A; intron MIR-148a), rs111299611(ins/del, 2KB Upstream variant of miR-148)	A (rs735316) and del (rs111299611) variants, result in lower MiR-148a levels and consequently higher *HLA-C* expression in individuals with intact miR-148 binding sites in the 3’UTR of *HLA-C.*	HIV, Crohn’s disease	(23)
*HLA-G*	rs1063320 (G>C, 3’UTR of HLA-G)	The presence of the C allele disrupts the binding of miR-148a to *HLA-G* resulting in higher expression of *HLA-G.*	Age-related macular degeneration, Asthma	(89–91)
		*In silico* studies showed that the presence of a C allele may also disrupt the binding of miR-19 and miR-218-2.		(92)
*HLA-G*	rs1707 (T>C, 3’UTR), rs1710 (C>G, 3’UTR) rs17179101 (A>C, 3’UTR), and rs17179108 (C>T, 3’UTR)	In silico analysis found that deletion of a 92bp region containing rs1707, rs1710, rs17179101, and rs17179108 may affect miRNA (miR-513a-5p, miR-518c*, miR-1262, miR 92a-1*, miR 92a-2*, and miR-661) mediated regulation of *HLA-G.*		(92)

The miR-148/-152 family (comprising miR-148a, miR-148b, and miR-152) plays a versatile role in various diseases by modulating the expression of important immune genes [reviewed in (93)]. Polymorphisms within the miR-148 family as well as certain HLA genes affect miR-148 family regulation of HLA and disease states (89, 90). For instance, Kulkarni et al. (2011) performed a sequence analysis of the 3’UTR of common *HLA-C* alleles and found polymorphisms within the binding sites of miR-148a, miR-148b, and miR-657 (24). These three miRNAs were found to have the highest predicted binding scores of the 26 miRNAs that are predicted to bind to HLA-C (24). Variants within the miR-148a/-148b binding site included rs67384697 (G>T), located at position 263 downstream of the stop codon, as well as the linked variants +259C/T, +261T/C, and +266C/T. Low-expressing *HLA-C* alleles allotypes (Cw*0702) with the G allele were found to be more stable due to miRNA binding compared to high-expressing *HLA-C* alleles (Cw*0602) marked with the T allele (24). The T allele disrupts the miRNA-148 a/-148b binding site, thus restricting the control miR-148a/-148b would otherwise have on the regulation of HLA-C. *HLA-C* expression was highest amongst individuals homozygous for the T allele, while those homozygous for the G allele had the lowest expression. Experimentally, miR-148b and miR-657 binding sites were not shown to drive *HLA-C* expression; however, the interaction between miR-148a and rs67384697 could be the causal variant for differential *HLA-C* expression (85). Rs67384697 was found to be in strong LD with rs9264942 which was shown to associate with *HLA-C* expression and control of HIV disease (25, 94–96). It is possible that rs67384697 is the causal variant responsible for controlling *HLA-C* expression and controlling HIV disease. High levels of *HLA-C* are associated with better control of HIV through more efficient antigen presentation to CD8+ T lymphocytes. In a cohort of 2,527 HIV-infected European Americans, HIV controllers were found to have a higher frequency of the T allele (rs67384697) and potentially have higher HLA-C expression and thus lower viral loads; while the G allele was significantly frequent in non-controller (24).

Sequence variation within the miR-148 binding site may not be the only explanation for differential levels of HLA-C. Kulkarni et al. (2013) also identified sequence variation within the *miR-148* gene which affected *miR-148* and subsequently *HLA-C* expression (23). Twenty-six polymorphic sites were identified in a 7.7kb region of the miR-148a gene and its flanking regions within 219 European American individuals (23). rs735316 (G>A) and rs111299611 (ins/del), were found to be in perfect LD and associated most significantly with miR-148 expression. Individuals homozygous for the wild-type del variant for rs111299611 and GG for rs735316 had higher levels of miR-148 compared to individuals homozygous for the mutant ins (rs111299611) and AA (rs735316) alleles. Lower *HLA-C* expression is expected among individuals who carry the wild type rs111299611 and rs735316 alleles (higher miR-148 expression) and have an intact miR-148 binding site within *HLA-C* 3’UTR and carry the wild type rs111299611 and rs735316 alleles (23). In a longitudinal cohort of 2,918 HIV-infected individuals, rs753516 genotypes were associated with HIV viral load in individuals with an intact miR-148 binding site and had no effect in individuals with a disrupted binding site. Furthermore, Kulkarni et al. (2013) showed that *HLA-C* expression had opposing effects on HIV and Crohn’s disease (23). Low miRNA-148 levels and high *HLA-C* levels are associated with better control of HIV, but a higher risk of Crohn’s disease. While high HLA-C expression increases the presentation of viral antigens to cytotoxic T cells and viral clearance, it could also lead to inappropriate activation of T cells, contributing to chronic inflammation in the gastrointestinal tract leading to Crohn’s disease. Kulkarni et al. (2013) further tested whether rs735316 is associated with the risk of Crohn’s disease. A meta-analysis found that rs735316 (AA) was associated with an increased risk of Crohn’s disease by lowering miR-148 expression and subsequently *HLA-C* expression in individuals with intact miR-148 binding sites located in *HLA-C* (23). This study demonstrates the need to study non-coding variants in disease-specific cases as they influence different diseases differently.

Predictive and functional analysis by Tan et al. (2007) showed that the G allele of rs1063320 (G>C) has a high affinity for miRNA binding as the predicted minimum free energy between G and miR-148/-152 family was highly stable compared to the C allele and miR-148/-152 family (88). These results were experimentally validated. Luciferase assay found that the activity of the G allele in the presence of the miRNA-148/-152 family was significantly diminished compared to the presence of the C allele or deletion of the whole miRNA target site. Furthermore, *HLA-G* levels were significantly reduced by miR-148a in JEG3 cells, which naturally express high levels of *HLA-G* and are homozygous G at rs1063320 (91). Similar results were observed in a study evaluating the role of statin treatment on miR-148/-152 family in asthmatic patients, *HLA-G* expression negatively correlated with miR-148/-152 levels in individuals homozygous for GG. However, there was no correlation between *HLA-G* and miR-148/-152 in individuals carrying CC or CG. Furthermore, the authors did not directly test the interaction between rs1063320, miR-148/-152, and HLA-G (89). On the contrary, Manaster et al. (2012) showed that rs1063320 does not influence the binding efficacy of the miR-148/-152 family to HLA-G as luciferase activity was repressed in the presence of both G and C in the 3’UTR (97). It is possible that the results differed due to different cell lines being used. For instance, ATP is essential for luciferase assays since endogenous ATP levels differ among cell lines, which may impact the results. *In silico* analysis supported previous findings that the G allele has a high affinity for miRNA binding as the minimum free energy between HLA-G and miR-148/-152 family was similar to that estimated by Tan et al. (2007) (88, 91). In addition, the presence of the G allele is a predicted target of miR-19 and miR-218-2 as the minimum free energies observed were similar to the miR-148/-152 family (92).

Eight polymorphic sites (rs1704 ins>del, rs1063320 G>C, rs1707 T>C, rs1710 C>G, rs17179101 A>C, rs17179108 C>T, rs9380142 A>G and rs1610696 C>G) have been observed within the 3’UTR of *HLA-G* including rs1063320 which was previously discussed (98). Rs1704 represents a 14bp ins/del variant that is in strong LD with rs1063320 and significantly associated with *HLA-G* expression levels and mRNA stability. The presence of the insertion variant marks reduced *HLA-G* levels (92). A subset of these transcripts is further processed by the deletion of 92bp originating in exon 8 and extending into the 3’UTR. This region contained rs1707, rs1710, rs17179101, and rs17179108 which may affect the binding of specific miRNAs (miR-513a-5p, miR-518c*, miR-1262, miR 92a-1*, miR 92a-2*, and miR-661) according to *in silico* studies (92).

The *HLA-DP* polymorphism, rs9277534 (A>G) is strongly associated with HBV persistence due to the strong effect it has on *HLA-DP* mRNA and surface protein levels. Individuals in the GG group have approximately twofold higher expression than individuals in either the AG or AA groups (26). The association of the G allele with high *HLA-DPB1* expression was also noted in acute graft versus host diseases and type 1 autoimmune hepatitis (99, 100). Computational assessment by Shieh et al. (2018) may explain the differences in expression. The A allele is present in low-expressing HLA-DPB1*04:01:01:01 (encoded by PGF B cell line); while high-expressing HLA-DPB1*03:01:01:01 (encoded by COX B cell line) transcripts contain the G allele. The 3’UTR of HLA-DPB1*04:01:01:01 was found to contain 27 predicted miRNA binding sites (101). Sixteen of these sites were also found in HLA-DPB1*03:01:01:01 and were targets of the same miRNAs. HLA-DPB1*03:01:01:01 had three additional miRNA binding sites and HLA-DPB1*04:01:01:01 contained one additional binding site that was not present in HLA-DPB1*03:01:01:01. Twenty and twenty-three miRNA binding sites were found in polymorphic regions of HLA-DPB1*03:01:01:01 and HLA-DPB1*04:01:01:01, respectively. The A allele of rs9277534 is associated with higher expression simply because it is targeted by a higher number of miRNAs (101). The authors further analyzed a publicly available data set of miRNA derived from 10 primary B cell samples (101, 102). Forty-four miRNAs were common in these 10 cell lines and the COX and PGF B cell lines. MiR-30e-3p was found to be the top candidate associated with rs9277534 as it is highly expressed and targets the A allele while disregarding the G allele (101, 102). However experimental validation and further functional analysis is necessary to identify if miR-30e-3p is indeed associated with rs9277534 or if one of the other predicted miRNAs is involved in rs9277534 mediated *HLA-DPB1* expression.

## The importance of the non-coding genome in HLA regulation and disease association

The HLA loci are strongly associated with a magnitude of diseases ranging from metabolic disorders to autoimmune conditions, cancers, and even infectious diseases. Due to HLA’s fundamental role in immune regulation, the HLA loci evolved to become the most genetically diverse gene region within humans (1, 2).

**Figure 3:**
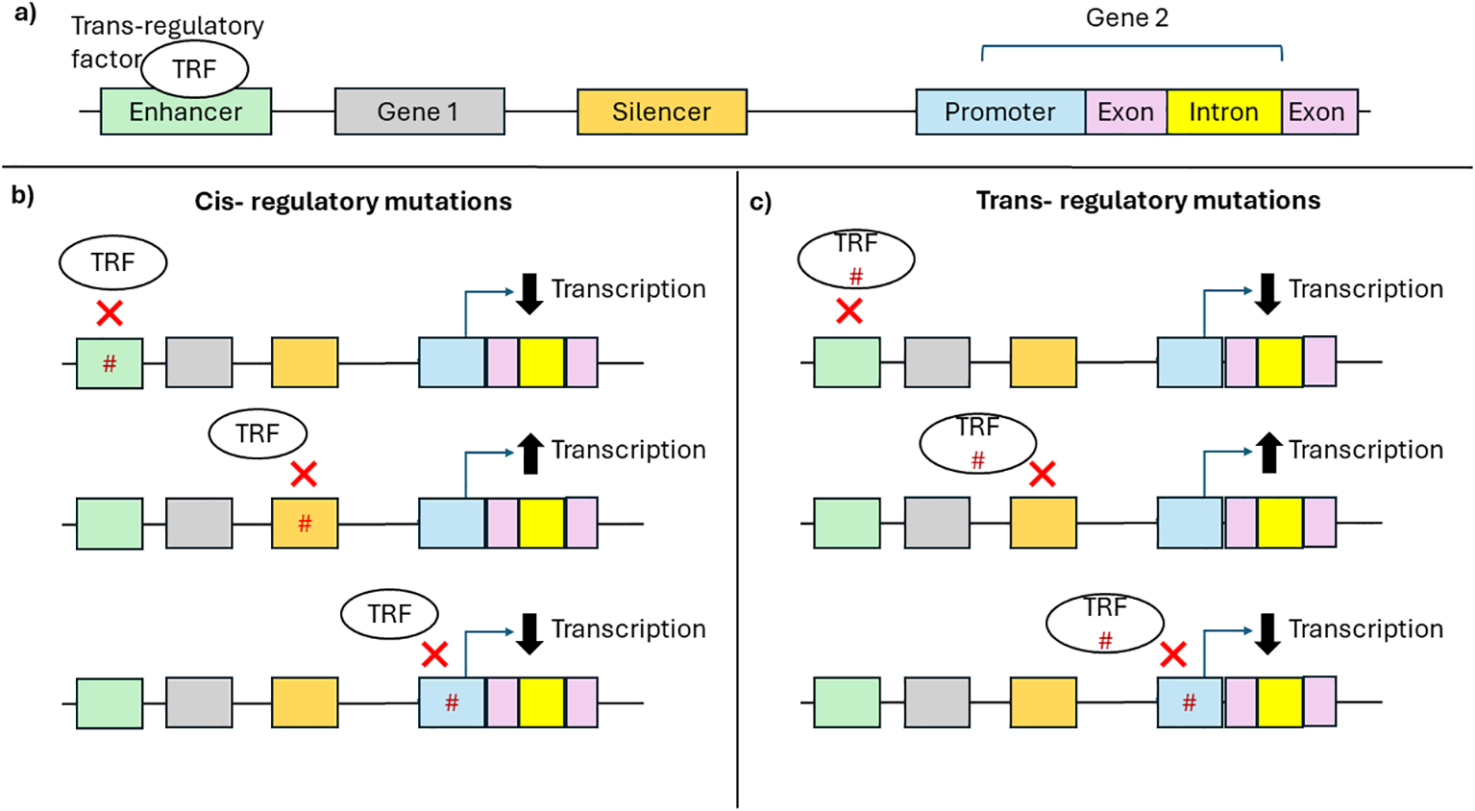
Effect of *cis-regulatory* mutations and *trans-regulatory* mutation of gene transcription. **(A)** Schematic representation of a gene region: cis-regulatory elements such as enhancer (green), silencer (orange), and promoter (blue); exons (pink), introns (yellow), and trans-regulatory factors such as transcription factors (white). **(B)** Mutations (hashtag) in *cis-regulatory* regions **(C)** or *trans-regulatory* factors can prevent the binding of *trans-regulatory* factors to *cis-regulatory* regions altering transcription.

It is well known that polymorphisms within the coding regions of HLA genes influence antigen presentation. The function of HLA molecules is also impacted by their expression levels which also critically impacts normal immunological functioning. Differential HLA expression is shown to be associated with various diseases and transplantation (31, 44, 58). Non-coding regions of the genome predominately control the regulation of HLA expression. The binding of trans-regulatory factors to cis-regulatory regions such as promoters, enhancers, silencers influences gene expression and UTRs (103). Motifs within cis-regulatory sites allow for the binding transcriptional machinery that turns transcription “on” or “off”. The promoter region is located around the transcriptional start site of a gene and contains several elements that facilitate the binding of transcription factors and the assembly of transcriptional machinery (103). While promoters act on neighbouring genes, enhancers contain motifs that sequester transcription machinery to induce transcription of distant genes. Silencers act to down-regulate gene transcription, either through the binding of transcriptional repressor proteins or by passively preventing the binding of transcription factors. Thus, mutations within the cis-regulatory regions or trans-regulatory factors can interfere with the binding of trans-regulatory factors, altering gene transcription and thus expression (**Figure 3**) (103). While promoters, silencers, and enhancers regulate expression at the transcriptional level, the 5’ and 3’ UTR regulate expression at a post-transcriptional level by influencing mRNA stability, mRNA localization and transport, and protein expression. The 5’UTR contains elements that impact the binding of ribosomes to the mRNA and subsequently the initiation of translation. The 3’UTR contains AU-rich elements and miRNA binding sites that regulate mRNA stability. Some miRNAs bind to the 5’UTR. The binding of miRNAs leads to mRNA degradation or inhibition of translation affecting protein levels. Thus mutations within the UTR can effect miRNA binding and alter the efficiency of protein translation (104).

We have discussed several studies in which non-coding variants lead to altered HLA expression and impact disease outcomes (23–25, 31, 40, 43, 44, 46, 48–55, 57–60, 70–78, 89–92). For instance, HLA-G plays a pivotal role in fetal-maternal immune tolerance and placental development. The -725 HLA-G variant is significantly associated with fetal loss. The G allele creates a CPG site near an IRF1 binding site. Methylation of the CpG site, prevents IRF1 biding resulting in lower HLA-G expression. Low HLA-G expression could lead to maternal immune activation against fetal antigens, leading to miscarriage (71). rs67384697G is present within low expressing HLA-C alleles as the G alleles allow for the binding of miR-148b, however, the presence of the T allele prevents miRNA-148b regulation of HLA-C and higher HLA-C expressions. rs67384697T has thus been associated with better control of HIV as the higher expression of HLA-C leads to more efficient presentation of HIV antigens to CD8+ T lymphocytes and viral clearance (24). Downregulation of HLA class I is a common escape mechanism for cancer cells from immunosurveillance and immunotherapy. rs41545520G creates a binding site for the transcriptional repressor, ATF3, leading to reduced HLA expression in nasopharyngeal carcinomas and consequently resulting in reduced presentation of tumour-associated antigens to CD8+ T lymphocytes and increased immune evasion (31). Somatic mutations add an extra layer to HLA diversity. In cancers, somatic mutations have been shown to contribute to changes in HLA expression and function supporting immune evasion (11, 105). Most studies investigating somatic mutations have been confined to coding regions, however, up to 96% of somatic mutations can occur within the non-coding genome and non-coding variants have been recognized as potential somatic catalysts and critic germline risk factors for cancer onset (106, 107). Thus, studying non-coding somatic mutations may also provide an improved understanding of the complexities of gene regulation and disease mechanisms.

Understanding mechanisms affecting the regulation of HLA expression is paramount for developing successful diagnostic and therapeutic approaches for different diseases (103). HLA-typing can be used to predict one’s susceptibility to disease and how an individual responds to the disease; however, it is an expensive and laborious process. In diseases where HLA expression is associated with severity or susceptibility to a disease, genotyping specific SNPs that play a role in regulating gene expression (such as those found in the non-coding genome) or determining the expression of certain HLA genes may be a more simple and cost-effective approach in predicting HLA-associated disease susceptibility and severity (108).

Altering HLA expression may also be important for certain therapeutic interventions. For instance, the onset of cancer is often associated with a decrease in class I HLA molecules. Several immunotherapeutic strategies have been designed to alter the tumor microenvironment. However, these strategies require normal HLA expression to be restored (109, 110). Targeting the non-coding genome either through genetic or epigenetic mechanisms will be essential for restoring HLA expression. According to ClinicalTrials.org, there are currently 509 and 332 clinical trials using miRNA and DNA methylation based therapy (111). This highlights the potential of epigenetic-based therapy that targets the most disease relevant region of the human genome.

Understanding the impact of the non-coding genome on HLA may further be used to optimize donor selection as well as broaden the donor pool for transplants. Hematopoietic stem cell transplantation is used in the treatment of hematological cancers and neoplastic disorders. It requires stringent matching of classical HLA class I and II alleles (HLA-A/-B/-C/-DRB1) between donor and recipient (112). Individuals requiring a transplant have a 30% chance of finding a first degree relative that has at least three matching HLA alleles (112). If a related donor cannot be found, an unrelated donor with a single mismatch can be used. This is however associated with a higher rate of mortality within a year (113). Furthermore, individuals from rare ethnic groups have a smaller chance of finding a suitable donor. One way of expanding the donor pool is by eliminating one of the HLA genes. Several studies have found HLA-A to be the most suitable candidate for elimination. Targeting of the non-coding genome using CRISPR-Cas9 and zinc finger nuclease was shown to effectively disrupt HLA-A (114, 115). This may improve the transplantation success involving unrelated individuals.

The importance of the non-coding genome in HLA regulation and pathophysiological states is extremely evident. More studies should be dedicated to exploring its potential for the design of diagnostic tools and therapeutic interventions.

## Ethnic-specific differences across HLA genes

It is well known that the HLA region has the highest number of polymorphisms in the human genome with the identification of 13,412 unique *HLA* alleles in humans. Migration history, racial admixture, environmental pressure, and selection pressure due to pathogen exposure have been suggested to be major contributing factors to the high level of variation observed in the *HLA* region (116, 117). Historically, malaria has been suggested to be a major driver in the evolution of African populations (118), while tuberculosis infection was the major driver in Western European populations (119). The high selection pressure exerted by these pathogens was mainly on genes involved in immune regulation, such as the *HLA* region. This may account for the differences in *HLA* allele frequencies observed between European and African populations. Africa has the highest level of *HLA* diversity in the world (120). Furthermore, it has been observed that regions that are further away from Africa have lower *HLA* diversity than those regions closer to the African continent. The high degree of diversity is most probably due to Africa being the source of modern humans and the high burden of infectious diseases (116, 120). Although Africa has the highest number of classical *HLA* alleles, with 3,141 classical *HLA* class I alleles being present in North Africa alone; the African region has lower *HLA* class II diversity compared to Europe, North America, and South America (116). The higher class I diversity in Africa may stem from the high prevalence of infectious diseases in the region; while auto-immune conditions which are usually linked to *HLA* class II alleles are more common in European and American populations (116).

The main mechanism contributing to the generation of diverse HLA alleles is point mutations (**Table 4**). Point mutations in the form of nucleotide substitutions can result in changes in amino acid sequence (non-synonymous) or no change of amino acid sequence (synonymous). Insertion or deletion of a nucleotide are also forms of point mutations that can change the reading frame creating a premature stop in translation. These point mutations can create alternative splice sites diversifying mRNA transcripts generated (121). For instance, splicing of exon 3 of HLA-G gives rise to several HLA-G isoforms (122). The recombination events: gene conversion (donation of DNA segment from one chromosome to its homologs), and crossing-over (bidirectional exchange of DNA between to homologous chromosomes), also contribute to HLA diversity. Recombination allows for the intergenic or intragenic exchange of nucleotide sequences between two chromosomes. For example, the HLA-B*53:44 allele was generated by an intralocus gene conversion between exon 3 of HLA-B*38 or HLA-B*39 and exon 2, part of exon 3 and 4 of HLA-B*53:01:01 (123). The HLA-C*07:294 allele was created through the intergenic crossing over of nucleotide sequence between exon 3 HLA-C*07:27:02 and HLA-B alleles such as HLA-B*07:02:01(exon 3) (121).

**Table 4:** Minor allele frequency of HLA non-coding variants in European, Asian and African populations.

		Minor allele frequency		
HLA gene	SNP	European	Asian	African
**HLA-A**	rs41545520	0.02	0	0
	rs9260102 (C>A)	0.096	0.156	0.18
	rs12202296 (T>C)	0.35	0.23	0.33
	rs41272547 (C>A)	0.015	0.018	0
	rs1061235 (C>T)	0.025	0.055	0.074
	rs9260118 (T>C	0.524	0.59	0.367
**HLA-C**	rs2395471 (G>A)	0.41	0.43	0.53
	rs2524094 (G>A)	0.57	0.59	0.48
	rs10657191 (AGAAG> AGAAGAAG)	0.51	0.56	0.65
	rs9264942 (T>C)	0.37	0.42	0.30
	rs2395471 (G>A)	0.40	0.43	0.53
	rs67384697 (G>T)	0.55	0.47	0.49
	rs735316 (G>A)	0.34	0.02	0.07
**HLA-G**	rs2395471 (G>A)	0.44	0.43	0.53
	rs915670 (C>G)	0.29	0.13	0.26
	rs9380142 (A>G)	0.32	0.24	0.19
	rs371194629 (ATTTGT> ATTTGTTCATGCCT)	0.23	0	0
	Rs1063320 (G>C)	0.48	0.73	0.59
	rs1707 (T>C)	0.85	0.99	0.90
	rs1710 (C>G)	0.48	0.73	0.60
	rs17179101 (A>C)	0.05	0.18	0.01
	rs17179108 (C>T)	0.10	0.19	0.11
***HLA-DRB1***	rs9271597 (T>A)	0.38	0.40	0.32
	rs9271600 (T>G)	0.24	0.05	0.02
***HLA-DQA1***	rs9271601 (A>T)	0.37	0.42	0.31
**HLA-DPA1**	rs3077 (A>G)	0.17	0.63	0.49
	rs2395309 (A>G)	0.16	0.55	0.43
	rs2301220 (C>T)	0.19	0.68	0.46
	rs1431403 (T>C)	0.28	0.60	0.54
	rs9277341 (T>C)	0.31	0.82	0.64
**HLA-DPB1**	rs9277535 (A>G)	0.24	0.57	0.24
***HLA-DQA1***	rs2187668 (C>T)	0.12	0.05	0.06
	rs28383345 (G>A)	0.12	0.09	0.09
	rs36173887 (A>G)	0.10	0	0.07
	rs114929610 (C>A)	0.03	0.11	0.11
***HLA-DQA2***	rs6906021 (T>C)	0.47	0.51	0.50
***HLA-DQB1***	rs1063355 (C>A)	0.60	0.61	0.52
***HLA-DRB1 and HLA-DQB1***	rs9275596 (C>T)	0.64	0.83	0.65
***HLA-DRB1/HLA-DRB6***	rs9271573 (A>C)	0.55	0.47	0.49

*HLA* polymorphisms and alleles are observed at different frequencies in different populations. Certain *HLA* polymorphisms may be found in certain populations and may not even exist in other populations (117). For instance, within the *HLA-A* locus, the HLA-A*02 family is the most diverse allele family consisting of 31 known alleles. HLA-A*02 alleles are determined by a combination of specific SNPs found within the coding region of HLA-A. Unlike other HLA allele families, HLA-A*02 is frequent in all ethnic groups, however, the frequency of HLA-A*02 alleles is different in various populations (124). A USA-based study evaluated the frequencies of HLA-A*02 subtypes across different ethnic groups positive for HLA-A*02. HLA-A*02:011 was predominantly found in Caucasian (~95.7%) and Native American (94.3%) populations whereas HLA-A*02:011 was found in 59% of African Americans, and 50% of Chinese. Interestingly, certain HLA-A*02 alleles were not present in some ethnic groups. HLA-A*02:02 (25.8%) and HLA-A*02:05 (12.9%) were common in African Americans but no present in Caucasians, Pacific Islanders, and Chinese populations. HLA-A*02:03 and HLA-A*02:07 were found only in the Chinese population (124). Van Rensburg et al. (2021) showed that there were no common predominant HLA alleles between South African Black and Caucasian populations. HLA-A*30:01, HLA-B*58:02, HLA-C*06:02, and HLA-DRB1*13:01 were shown to be the predominant alleles in the black population, while HLA-A*02:01:01, HLA-B*07:02:01, HLA-C*07:01, and HLA-DRB1*03:01 was shown to be predominant in South African Caucasians. HLA-A*30:01:02, HLA-A*30:02:02, HLA-A*68:27, HLA-B*42:06, and HLA-B*45:07 were also shown to be unique to Black South Africans (125). Ethnic differences in the frequency of non-coding HLA SNPs can also occur. The National Center for Biotechnology Information created the single nucleotide polymorphism database (dbSNP) which contains allele frequency data for two thousand individuals across different populations. Using dbSNP, we identified the frequency of non-coding HLA SNPs (discussed in this review) within European, African, and Asian populations. For many of the SNPs, especially within class II genes, the frequency differed between ethnic groups. For instance, the minor allele of rs9277341 was found in 31% of Europeans, 82% of Asians and 64% of Africans. In some cases the SNP was absent in certain populations such as in the case of rs371194629 which is present in 23% of Europeans but absent in Asian and African populations.

The extensive differences in *HLA* allele diversity are associated with disease risk and progression in different ethnic groups (126, 127). For example, ethnic-specific differences regarding HLA were observed in Hepatitis C infection. Hepatitis C viral clearance correlated with HLA-DQB1*03, HLA-DRB1*11, and HLA-DRB3*02 in Caucasians, however, this was not observed in African individuals (126). Regarding ulcerative colitis, HLA-DRB1^*^15:03 is shown to be a risk allele for African American population, whereas HLA-DRB1^*^15:01, is a risk allele in Caucasian populations, where it is more frequent. Likewise, HLA-DRB1*09:01 is associated with ulcerative colitis in the Korean and Japanese populations (127). Ethnic-specific responses to disease are often associated with HLA coding regions as it induces changes in peptide binding; however, variation affecting HLA expression may also be associated with different responses to disease across different ethnicities.

Differences in *HLA* frequency across populations are also linked to adverse drug reactions experienced in certain populations (128). For instance, Abacavir is used in the treatment of HIV. Peptide fragments or metabolites of Abacavir complex with HLA-B*57, which activates T cells resulting in hypersensitivity to the drug (129). Abacavir hypersensitivity is more likely to affect Caucasians than Africans and Asians since the presence of HLA-B*57 is more frequent in Caucasians (129). Similarly, Carbamazepine is used in the treatment of neurological disorders such as epilepsy and bipolar. Carbamazepine hypersensitivity was shown to be more common in Asian populations compared to European populations (128). Asian populations with high frequency of HLA-B*15:02 such as Han Chinese, Malaysians, and Thai have been shown to associate with Carbamazepine hypersensitivity (128). HLA-B*15:11 and HLA-A*31:02 was shown to associate with Stevens–Johnson syndrome (SJS), and toxic epidermal necrolysis carbamazepine-induced hypersensitivity in Japanese and Koreans (128).

These marked differences in the distribution of *HLA* alleles across populations and ethnic groups make global treatment and vaccine strategies difficult to implement as certain groups may respond well, while others may not. This ethnic-specific bias requires ethnic-specific solutions to avoid disparities in global health care.

## Guidelines for future HLA studies

Since its discovery, the HLA region has been one of the most well-studied gene regions in the human genome. However, there is still a lot to uncover about this region. In this section, we provide guidelines that should be considered in future studies involving the HLA region.

While variation within the coding region has been well studied as it affects peptide binding, the non-coding regions of *HLA* genes remain understudied (130, 131). The non-coding genome accounts for more genetic diversity than coding regions and SNPs within this region can affect the expression of specific HLA genes which may have disease consequences (18, 23–26). Yet from our literature search, only 23 studies evaluated the non-coding region of various HLA genes and their association with HLA expression and/or disease outcomes. To our surprise, no SNPs within the non-coding region of HLA-B were shown to associate with the regulation of HLA-B. This is surprising as HLA-B is the most polymorphic HLA gene, consisting of approximately 3000 different alleles (38). Furthermore, most studies discussed in this review had only investigated the effect non-coding variants had on *HLA* gene expression. Gene expression does not always correlate to protein levels, as approximately 40% of protein variance within mammalian cells correlates with mRNA abundance. Regarding, HLA expression, Aguair. V.R.C., et al. (2023) did find that HLA-C surface expression did correlate with mRNA expression (qPCR: r=0.59 and RNA-seq: r=0.67) (132). The discrepancy may be due to the multitude of regulatory events that occur between transcription and translation, such as alternative splicing or silencing by miRNAs (133). Furthermore, non-coding variants that occur within introns, 5’ and 3’ UTR may impact post-transcriptional processes such as splicing, polyadenylation, cleavage, ribosome binding, and assembly, thus impacting mRNA translation to protein. Therefore, it is important to evaluate whether these non-coding variants have an impact on HLA protein levels (134). We suggest that more research should be conducted on evaluating the variation within the non-coding genome and the functional consequences it may have on *HLA* gene and protein expression and disease outcomes.

Epigenetic studies provide valuable insight into the regulation of *HLA* genes across different diseases. Due to the limited number of studies evaluating epigenetic mechanisms regulating HLA, this review only discussed the epigenetic mechanisms DNA methylation and miRNAs. However, histone modifications and long non-coding RNAs (lncRNA) are also involved in HLA regulation. For example, the lncRNA, HOTAIR induces HLA-G expression by silencing the miR-152 in gastric cancer (135). Acetylation of lysine residues on H3 and H4 was also shown to induce HLA-G expression by promoting a permissive chromatin state (136). Unlike genetic factors, epigenetic factors are reversible and can be used to influence gene regulation without resulting in permanent changes (27). Epigenetic factors can increase the expression of low expressing *HLA* genes to improve pathogen clearance or decrease the expression of high expressing alleles to prevent hyperactive immune responses. Thus, understanding the epigenetic mechanisms involved in regulating HLA may have therapeutic advantages.

Although HLA diversity is the highest in African populations, only a limited number of African ethnic groups have been HLA-typed. African countries such as Angola, Lesotho, Malawi, Namibia, and Swaziland do not have any HLA data available (120). Where *HLA* data is available for African countries, this data is usually derived from disease association studies which is not a true reflection of the general population. Therefore, there is limited understanding of the HLA diversity observed in African populations and the associations it may have with diseases and vaccine development (120). Thus, to fully elucidate the extent of diversity within the HLA loci, more studies should evaluate the HLA diversity from the general population in the African region. Furthermore, resources similar to HLA-net which focuses on HLA diversity in Europeans and its application in population genetics, transplantation, and epidemiology may be used to improve donor selection, population studies, and disease association studies in Africa (120, 137, 138).

Lastly, to thoroughly evaluate HLA diversity, high resolution techniques such as next generation sequencing should be used. We previously discussed that HLA-A*02 is found in all ethnic groups but the frequency of HLA-A*02 subgroups differs amongst different ethnic groups. High resolution HLA typing will provide a more in-depth understanding of HLA diversity in specific ethnic groups (124, 139).

In summary, we suggest that future HLA-based studies should focus on: (I) the non-coding region of HLA, (II) epigenetic regulation of HLA, (III) understudied populations, and (IV) use high resolution typing. This will greatly improve our understanding of this complex loci.

## Concluding remarks

With approximately 11,000 distinct protein variants, it is well known that the HLA loci are the most genetically diverse region. While genetic variants within the coding region have been long known as an important contributor to complex pathophysiological states, recent studies have identified functional variants within the non-coding regions of HLA. Furthermore, these genetic variants interact with epigenetic mechanisms further contributing to the complexity of HLA regulation. HLA association becomes even more complicated when we take into consideration the high level of diversity between different ethnic groups. Fully understanding the complexity of the HLA regions will result in improving the development of diagnostic approaches, therapeutic strategies, vaccine design, and transplantation procedures; improving overall global health. This review highlighted the importance of the non-coding genome concerning HLA regulation and different disease states. In this review, we: (i) discuss genetic non-coding variants that affect HLA expression, (ii) link genetic variation with epigenetic regulation of HLA genes, (iii) highlight the importance of the non-coding genome in HLA regulation and disease association, (iv) evaluate ethnic-specific differences across HLA genes and provide guidelines for future HLA studies.
